# Precise regulation of the relative rates of surface area and volume synthesis in bacterial cells growing in dynamic environments

**DOI:** 10.1038/s41467-021-22092-5

**Published:** 2021-03-30

**Authors:** Handuo Shi, Yan Hu, Pascal D. Odermatt, Carlos G. Gonzalez, Lichao Zhang, Joshua E. Elias, Fred Chang, Kerwyn Casey Huang

**Affiliations:** 1 Department of Bioengineering, Stanford University, Stanford, CA USA; 2 Department of Cell and Tissue Biology, University of California, San Francisco, San Francisco, CA USA; 3 Department of Chemical and Systems Biology, Stanford University School of Medicine, Stanford, CA USA; 4 Chan Zuckerberg Biohub, Stanford, CA USA; 5 Department of Microbiology and Immunology, Stanford University School of Medicine, Stanford, CA USA

**Keywords:** Cell growth, Bacteria, Bacteriology, Cellular microbiology

## Abstract

The steady-state size of bacterial cells correlates with nutrient-determined growth rate. Here, we explore how rod-shaped bacterial cells regulate their morphology during rapid environmental changes. We quantify cellular dimensions throughout passage cycles of stationary-phase cells diluted into fresh medium and grown back to saturation. We find that cells exhibit characteristic dynamics in surface area to volume ratio (SA/V), which are conserved across genetic and chemical perturbations as well as across species and growth temperatures. A mathematical model with a single fitting parameter (the time delay between surface and volume synthesis) is quantitatively consistent with our SA/V experimental observations. The model supports that this time delay is due to differential expression of volume and surface-related genes, and that the first division after dilution occurs at a tightly controlled SA/V. Our minimal model thus provides insight into the connections between bacterial growth rate and cell shape in dynamic environments.

## Abstract (web summary)

Bacterial cells actively change their size and shape in response to external environments. Here, Shi et al. explore how cells regulate their morphology during rapid environmental changes, showing that the characteristic dynamics of surface area-to-volume ratio are conserved across genetic and chemical perturbations, as well as across species and growth temperatures.

## Introduction

In their natural habitats, bacterial cells constantly face dynamic environmental conditions. To survive, cells alter their physiology to cope with stresses such as nutrient depletion, chemical inhibition, and temperature shifts. During stressful conditions, cells alter their gene expression profiles, often slowing down growth and proliferation and instead allocating limited resources to genes critical for survival^1^. While certain genetic perturbations do not have observable effects in fast-growing cells, they cause death in stressed conditions^2^ or impair survivability when cells resume growth after the environment becomes favorable again^3,4^, highlighting the unique physiological challenges posed by dynamic environments.

Cell shape is intrinsically linked to physiology. During steady-state growth, fast-growing cells in nutrient-rich media adopt larger volumes compared to isogenic cells in minimal media^5^, and systematic tuning of growth rate via medium composition dictates steady-state cell size^6,7^. Gene expression is also modulated by steady-state growth rates: faster-growing cells tend to have a higher fraction of their proteome devoted to ribosomes, addressing the need for rapid protein synthesis^8,9^. In a batch culture, cell shape can undergo transitions in both cell width and length within several minutes^10,11^, in part as cells adapt their transcriptional program to the new medium and also because the growth of cells can alter the medium composition through nutrient depletion and waste production. Previously, a top-down flux-balance model accurately depicted the kinetics of gene expression and growth in *Escherichia coli* cells under nutrient shifts^12^, but it remains unclear how these environmental and gene expression changes are transduced into cell-shape changes, and whether cell shape is actively optimized in a dynamic environment or is simply a passive outcome of cellular physiology.

In most bacteria, cell shape and size are dictated by the cell wall, a rigid network of peptidoglycan^13,14^. To grow and divide, cells synthesize new peptidoglycan precursors in the cytoplasm, which are then transported to the periplasm and inserted into the expanding cell wall^14^. In rod-shaped bacteria such as *E. coli*, cell elongation and division are regulated by distinct machineries. The actin homolog MreB dictates the insertion pattern of new peptidoglycan material along the cylindrical cell body^15^, which elongates the cell and maintains steady-state cell width^16^. Cell division is regulated by FtsZ, a tubulin homolog that localizes to the mid-cell and forms a ring-like structure prior to division, which then constricts and guides septum formation^14^. Chemical or genetic perturbations to the elongation or division machinery alter cell-shape homeostasis through modified patterns of cell wall synthesis^10,11,16–19^. The extent to which such perturbations disrupt the ability of bacterial cells to adjust to new environments could provide insight into the cellular processes responsible for shape adaptation.

While cell width and length are thought to be regulated by distinct molecular mechanisms, previous studies have indicated that they are somewhat inter-connected. In a non-essential gene knockout library, the mutants collectively exhibited variation in both mean cell width and length, with a positive correlation between width and length^20^. Similarly, single point mutations in the MreB protein can alter both width and length^21,22^, yet a large library of MreB mutants were found to all occupy a specific region of the space of cell geometries during growth in LB in which both wider and thinner mutants had longer mean lengths compared to wildtype^11^. Therefore, cell width and length seem to be regulated by an upstream process that unifies the two aspects of cell shape. In a previous study, it was shown that the regulation of surface area to volume ratio (SA/V) is such a process upstream of cell width and length determination: switching cells at steady state to a condition in which only cell wall (i.e., surface area) synthesis is partially inhibited increased both cell width and length, which lowers SA/V^23^. Similarly, SA/V changes during the different stages of growth, with log-phase cells having lower SA/V compared to stationary phase^23^; those measurements were performed in highly controlled microfluidic chambers, and cells took tens of minutes to several hours to fully adapt to the new steady-state morphology after the near-instantaneous switch in media. The more continuous changes that cells undergo in a dynamic environment such as that encountered in a batch culture have yet to be fully understood, particularly from the perspective of cellular geometry.

In this study, based on precise and frequent experimental measurements of cellular dimensions and growth rates of a batch culture constantly experiencing nutrient compositional changes and waste accumulations, we develop a model that quantitatively predicts SA/V dynamics. Our model predicts a time delay between surface area and volume synthesis adaptation, and that cells outgrowing from stationary phase will always experience a period of active width increase due to optimal resource allocation to volumetric growth. This model focuses on global resource constraints rather than specific molecular machineries, and therefore is broadly applicable to other microbial batch cultures. Indeed, we found that the qualitative SA/V dynamics we observed are universal across rod-shaped microbial species and growth conditions. With only a single free parameter, our time-delay model predicts the SA/V changes due to perturbations in cell-wall synthesis or protein translation. Our work highlights the ability of bacterial cells to rapidly respond to changing environments by modifying their physical growth.

## Results

### Cell growth and dimensions undergo complex changes in a batch culture

**Fig. 1:**
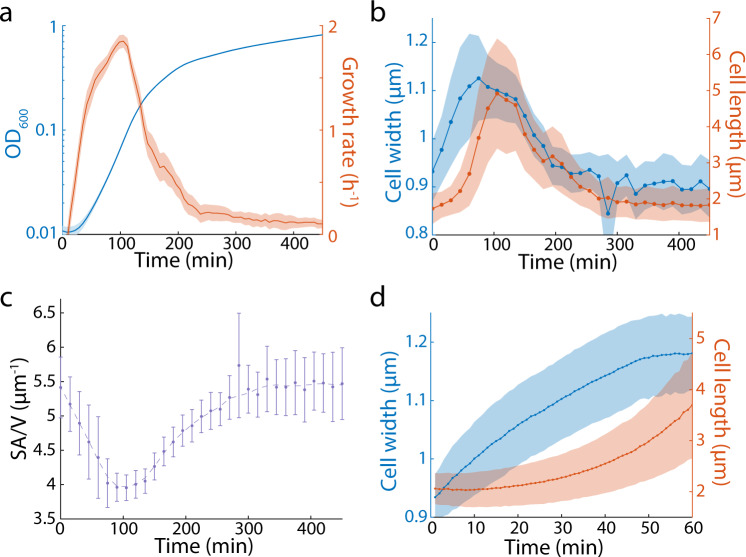
Cell growth and dimensions undergo complex changes in a batch culture. **a** Growth curve and the corresponding growth rate of an *E. coli* MG1655 batch culture after diluting the overnight culture 1:200 into fresh LB. Growth rate peaks at ~1.5 h post dilution, and then slowly decreases. Data points and the corresponding shaded areas are mean ± standard deviation (S.D.) for *n* = 12 replicates. **b** Shape growth curves (cell width and length as a function of time) for the same culture in **a**. Cell width starts to increase earlier compared to length. Data points and the shaded areas are mean ± S.D. for *n* > 200 single cells. **c** SA/V as a function of time for the same culture in **b**. SA/V decreased as cells resume growth, and then slowly increased back to the initial value when cell growth slowed down. Data points are mean ± S.D. with *n* > 200 single cells, and the dashed line is smoothed as a guide to the eye. **d** Single-cell time-lapse imaging of stationary-phase MG1655 cells diluted onto an agarose pad containing fresh LB. Each cell exhibited similar width and length dynamics as in the bulk culture in **b**. Solid lines and the corresponding shaded areas are mean ± S.D. with *n* = 146 single cells. Source data are provided as a Source Data file.

In previous studies, we showed that as *E. coli* cells transition from stationary phase to log phase and back to stationary phase in a batch culture, cellular dimensions vary along with the instantaneous growth rate^10,11^. After a 1:200 back-dilution of an overnight culture grown in LB into fresh LB, cells resumed growth and reached their maximum growth rate after ~1.5 h, after which growth rate gradually slowed down to approximately zero by ~4–5 h (Fig. 1a). To validate our previous measurements, we extracted a small sample of cells every 15 min and quickly spotted them onto agarose pads for single-cell imaging and quantification (Methods). Mean cell length increased only slightly in the first 0.5 h. The peak in bulk growth rate at 1.5 h corresponded with the peak in mean cell length across the population (Fig. 1b), which increased by ~3-fold relative to stationary-phase cells. Mean cell width increased linearly within minutes after dilution, and reached its maximum after ~1 h, increasing by ~25% relative to stationary-phase cells (Fig. 1b). Since both length and width initially increase, the surface area-to-volume ratio (SA/V) decreased over this time; SA/V reached its minimum at approximately the same time as the peak in growth rate and mean length (1.5 h; Fig. 1c). After 1.5 h, the dynamics of length, width, and SA/V were more gradual, with all quantities reaching plateaus by 5 h. We term these measurements of cell dimensions throughout a passage cycle as a “shape growth curve,” by analogy to absorbance measurements. Using such measurements, we can accurately capture single-cell shape dynamics in a liquid batch culture over extended time periods.

To confirm that the observed changes in cellular dimensions and SA/V were not due to artifacts of sampling, we performed time-lapse imaging by diluting and spotting stationary phase cells onto an agarose pad containing fresh medium, and tracked the same cells for 1 h as they resumed growth on pads. The growth rates of these cells mimicked a population grown in liquid culture, and their shape dynamics recapitulated shape growth curves (Fig. 1d). In particular, each single cell increased in width within several minutes after they were placed on the agarose pad with fresh medium, while length did not increase noticeably until 20–30 min later (Fig. 1d). Therefore, our shape growth curves (Fig. 1b, c) indeed reflect the morphological changes for each single cell seen in batch culture.

### A time-delay model explains the relative dynamics of surface area and volume synthesis in batch cultures

**Fig. 2:**
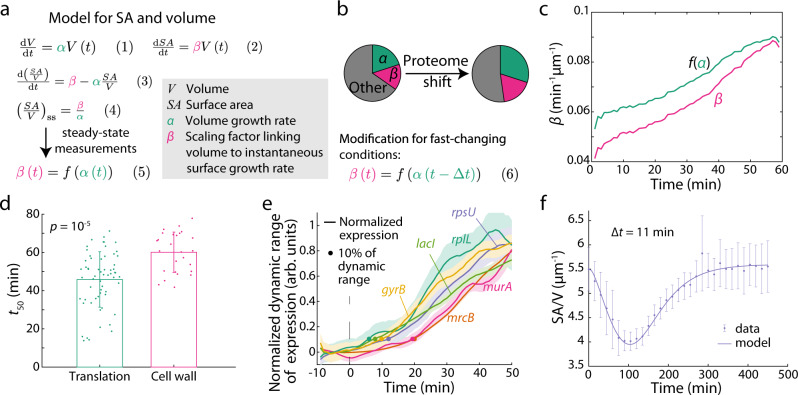
A time-delay model explains the relative dynamics of surface area and volume synthesis in batch cultures. **a** A conceptual model for SA/V regulation. The synthesis rates of both surface area and volume scale with current cell volume (Eq. 1, 2), predicting that the time derivative of SA/V depends on *α*, *β*, and the current SA/V (Eq. 3). Steady state SA/V is therefore dictated by the ratio between *β* and *α* (Eq. 4). Using previous experimental steady-state SA/V measurements, *β* can be parameterized as a function of *α* in steady state (Eq. 5). **b** Given that changes in growth rate (*α*) are accompanied by shifts in proteome composition, our model assumes that the shift is more heavily weighted toward cytoplasmic components than surface area components, thereby causing a delayed change in *β*, which can be approximated by introducing a constant time delay in the function relating *β* to *α* (Eq. 6). **c** From the single-cell data in Fig. 1d, the experimentally measured *β* exhibited a delay of ~10 min compared to the steady-state *β* determined by Eq. 5 (i.e., *f*(*α*)). **d** The rate of protein abundance change from our proteome measurements (Methods) was approximated by *t*_50_, defined as the time required to reach 50% of the difference between stationary phase and log phase abundances. The *t*_50_ of proteins involved in translation was ~10 min shorter compared to proteins involved in cell wall biosynthesis. *p* = 10^-5^, one-tailed Student’s *t* test. Each dot is a protein (*n* = 61 translation genes and *n* = 26 cell wall genes), and bars are mean ± S.D. for each group. **e** Protein expression levels were measured using GFP fused to the respective promoters and normalized from 0 to 1 within their dynamic range during the time-lapse. The cytoplasmic proteins increased in expression ~10 min earlier than the cell-wall synthesis proteins. Dots represent the time points at which expression had increased by 10%. Data are mean ± standard error of mean with *n* > 100 cells. **f** Fitting our time-delay model to the experimental data in Fig. 1c with Δ*t* = 11 min yielded an excellent fit. Data points are mean ± S.D. with *n* > 100 single cells. Source data are provided as a Source Data file.

Previous work showed that if surface area synthesis, like volume synthesis, is dependent on cell volume (rather than surface area), SA/V will equilibrate at a steady-state value corresponding to the ratio of surface and volume synthesis rates^23^ (Fig. 2a). Here, “volume synthesis” is represented by the synthesis of cytoplasmic proteins. Our measurements for cells transitioning out of and into stationary phase are clearly not at steady state as growth rate is constantly changing, and indeed mean SA/V varied by ~25%. If we extend the exponential growth laws^23^ for volume *V*, $\frac{{{\mathrm{d}}V}}{{{\mathrm{d}}t}} = \alpha V\left( t \right)$, and surface area *SA*, $\frac{{{\mathrm{d}}SA}}{{{\mathrm{d}}t}} = \beta V\left( t \right)$, to cover non-exponential growth through time-dependent functions $\alpha \left( t \right)$ and $\beta \left( t \right)$, we can derive an equation for the dynamics of SA/V (Fig. 2a):

3 $$\frac{{{\mathrm{d}}\left( {\frac{{{SA}}}{V}} \right)}}{{{\mathrm{d}}t}} = \frac{1}{V}\frac{{{\mathrm{d}SA}}}{{{\mathrm{d}}t}} - \frac{{{SA}}}{{V^2}}\frac{{{\mathrm{d}}V}}{{{\mathrm{d}}t}} = \beta \left( t \right) - \alpha \left( t \right)\frac{{{SA}}}{V}$$

At steady state, $\frac{{{\mathrm{d}}\left( {\frac{{{SA}}}{V}} \right)}}{{{\mathrm{d}}t}} = 0$, and therefore $\frac{{{SA}}}{V} = \frac{\beta }{\alpha }$ (4). Previous studies have measured these steady-state SA/V values across media that support different growth rates *α*, showing that steady-state SA/V was approximately linear (with negative slope) as a function of *α*^6^. Hence, we approximated *β* as a hyperbolic function of *α* based on steady-state SA/V measurements (Methods), providing an empirical relationship $\beta \left( t \right) = f\left( {\alpha \left( t \right)} \right)$ (5). With our measured value of initial SA/V at *t* = 0, the dynamics of *α* (calculated from optical density readouts), and the function *f*, we obtained a prediction for the SA/V dynamics during a shape growth curve (Supplementary Fig. 1a), and found that the model poorly predicted our experimental measurements. Specifically, the model predicted a slower initial decrease in SA/V, and the minimum value was higher and occurred at a later time than our experimental measurements. The final SA/V value never recovered to the initial value, even though the growth rate *α* was 0 at the beginning and end. Thus, the model based on a quasi-steady state assumption does not capture some key factor(s) contributing to SA/V changes during batch culturing.

Studies focused on the *E. coli* proteome have shown that conditions supporting higher growth rates require reallocation of protein synthesis toward ribosomes^8,9^. We hypothesized that the transition from stationary phase to log-phase growth would require a similar shift in proteome composition more heavily weighted toward cytoplasmic components than surface area components (Fig. 2b), which could lead to different temporal dynamics between *α* and *β* contrary to the quasi-steady state hypothesis. To explore such a possibility, we analyzed our single-cell time-lapse trajectories (Fig. 1d) to measure *α* and *β* for each cell. From single-cell measurements of *α*, we calculated *f*(*α*), the expected value of *β* during steady-state growth at rate *α*. We found that *f*(*α*) increased more quickly than *β*, with a roughly constant time delay of ~10 min between the two curves (Fig. 2c).

We next sought to directly quantify the dynamics of *α* and *β* using proteomics. We extracted proteins from cells at multiple times during stationary-phase outgrowth, and analyzed protein-abundance dynamics (Methods, Supplementary Data 1). For each protein that increased in abundance during outgrowth, we fit its dynamics to a logistic curve, and defined *t*_50_ as the time at which the midpoint was reached. Proteins involved in translation had an average *t*_50_ of 46 ± 14 min (mean ± S.D.), while proteins involved in cell-wall biosynthesis had an average *t*_50_ of 60 ± 10 min (Fig. 2d), consistent with our inference that changes in *β* occur ~10 min slower than changes in *α*.

To confirm our proteome measurements, we utilized a library of *E. coli* strains with GFP reporting the expression from various promoters^24^ and directly quantified the transcriptional dynamics of genes encoding cytoplasmic and surface-related proteins as cells emerge from stationary phase. We tested two strains representing ribosomal proteins (P_*rplL*_-GFP, P_*rpsU*_-GFP), two representing other cytoplasmic proteins (P_*lacI*_-GFP, P_*gyrB*_-GFP), and two representing enzymes related to cell-wall synthesis (P_*mrcB*_-GFP, P_*murA*_-GFP). We loaded stationary-phase cells into a microfluidic device surrounded by the supernatant from the overnight culture, then switched to fresh LB medium and monitored single-cell gene expression. In all six strains, GFP levels increased during growth, signifying increased expression. Consistent with our model prediction, the promoters of cytoplasmic proteins increased expression faster than those of the cell-wall genes (Fig. 2e, Supplementary Fig. 1b, Supplementary Note 1). We quantified the expression dynamics by calculating *t*_10_, the time for each promoter to increase their relative expression by 10% of the dynamic range between the beginning and end of imaging. The promoters for cytoplasmic proteins had *t*_10_ = 10 min, whereas the promoters for cell-wall enzymes had *t*_10_ = 20 min, a delay of ∆*t* = 10 min. Therefore, our direct measurement of gene expression dynamics confirmed a ~10 min delay between *α* and *β*, supporting differential regulation of the proteome (Fig. 2b).

Motivated by the observation that changes in *β* are delayed compared to *α*, we modified our model in Eq. 3 by substituting the function between *α* and *β* to be $\beta \left( t \right) = f\left( {\alpha \left( {t - \Delta t} \right)} \right)$, where Δ*t* is a constant that characterizes the time delay between *β* and *α*. Fitting our experimental data with the time-delay model yielded almost perfect agreement with a time delay Δ*t* = 11 min (Fig. 2f). The Δ*t* from fitting SA/V is consistent with our direct measurements of protein abundances (Fig. 2d) and gene expression (Fig. 2e), further validating our hypothesis regarding the proteome shift (Fig. 2b). Our derivation assumes that both volume and surface area growth scale with volume (Fig. 2a), but the time-delay model still holds if growth scales with surface area (Supplementary Note 2). Thus, the minimal time-delay model with a single free parameter is able to almost entirely recapitulate the quantitative features of SA/V and transcriptional dynamics.

### Cell widening during exit from stationary phase is robust across environmental and genetic perturbations

**Fig. 3:**
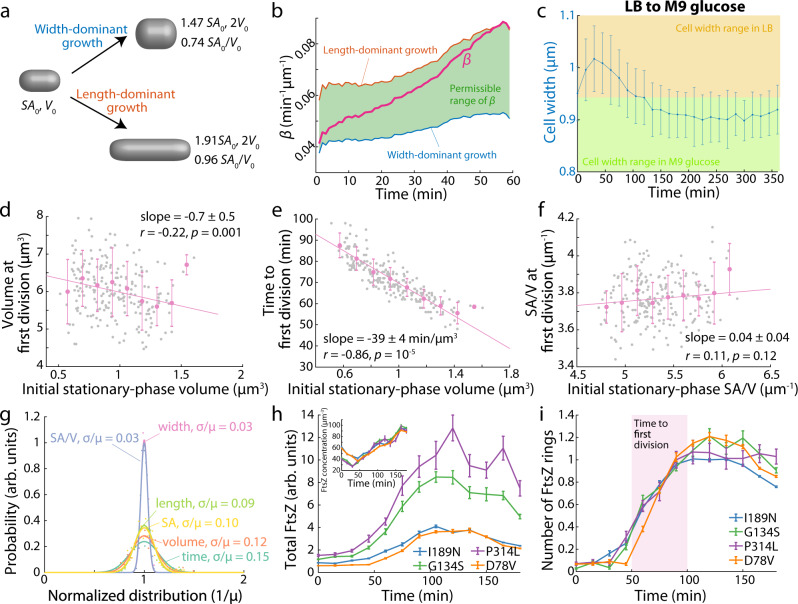
SA/V dynamics are correlated with the first cell division after stationary phase exit. **a** For a rod-shaped cell starting with surface area *SA*_0_ and *V*_0_, doubling its volume by expansion in width requires a 47% increase in surface area (top), whereas expansion in length requires a 91% increase in surface area (bottom). Therefore, an increase in width minimizes the surface area requirement for a given amount of volumetric growth. **b** Given the geometry and instantaneous growth rate (*α*) of cells in Fig. 1d, to maintain rod-like shapes, only a certain range of *β* values are permissible (green, Methods). The actual value of *β* started close to its minimal possible value, characterizing a widening-dominant growth mode; by 60 min, *β* reached its maximum possible value, transitioning to an elongation-dominant growth mode. **c** Dilution of stationary-phase cells grown in LB into M9 glucose caused an increase in cell width, despite the fact that cells continuously passaged in M9 glucose always had lower cell width than the LB-grown stationary-phase cells. Data are mean ± S.D. with *n* > 100 cells. **d**–**f** For cells exiting stationary phase, the volume at the first division was negatively correlated with the starting volume (**d**). The time to first division after stationary-phase exit was negatively correlated with the initial cell volume (**e**). The SA/V at the first division was largely constant and independent of the initial SA/V of stationary-phase cells (**f**). Gray dots are data points for *n* = 209 individual cells, and pink data points are binned mean and S.D. values, which were fit to a linear model. Data for slopes are mean ± s.e.m. *r,* Pearson’s *r*. *p*-values are from two-tailed Student’s *t* tests. **g** Normalized distributions of cell length, width, SA, volume, SA/V, and time at first division after stationary-phase exit. SA/V and width had by far the narrowest distributions. **h** FtsZ abundance was measured as total fluorescence intensity inside cells. The dynamics of FtsZ regulation were similar across mutants. FtsZ abundance did not change in the first ~50 min, then increased to a maximum at ~100 min. Inset: While larger cells tended to have lower SA/V and higher FtsZ levels, FtsZ concentrations were highly similar across shape mutants. Data points are mean ± s.e.m. with *n* > 200 cells. **i** All strains started without FtsZ rings and did not possess a ring until ~50 min post dilution, consistent with the onset of first divisions after stationary phase exit (**e**). By 100 min, virtually all cells had ~1 FtsZ ring. Data points are mean ± s.e.m. with *n* > 200 cells. Source data are provided as a Source Data file.

Our ability to fit the complex SA/V dynamics during batch culture with a simple model involving only the introduction of a time delay to the steady-state relationship between *β* and *α* suggests a simple picture of the initial stages of growth: as cells emerge from stationary phase, they devote more resources to synthesizing cytoplasmic components such as ribosomes than to surface components such as the cell wall. While the cell still must expand to allow space for these new cytoplasmic components, it does so with a minimal amount of surface growth by expanding predominantly in width rather than length, as volume scales approximately quadratically with width but only linearly with length. Indeed, in a typical rod-shaped cell, a two-fold change in volume requires only a 47% increase in surface area if cells only increase in width, but 91% if cells increase only in length (Fig. 3a, Methods). To test this reasoning, from our single-cell time-lapse results (Fig. 1d), we directly calculated the possible range of *β* at each time point using the observed width, length, and growth rate *α* (Methods). At *t* = 0, the measured *β* was close to its minimal possible value, characterizing a widening-dominant growth mode. By *t* = 60 min, the measured *β* approximately reached its maximum under those growth conditions, consistent with the canonical elongation-dominant growth mode (Fig. 3b). Thus, during outgrowth from stationary phase, cells transition their growth mode from widening to elongation.

To further validate that the initial increase in width during outgrowth from stationary phase (Figs. 1b, d and 3b) is governed by the delay between *α* and *β*, rather than determined solely by the current nutrient condition after back dilution, we took stationary-phase cells grown in LB and diluted them into M9 glucose medium. Cells grown in LB always had widths larger than 0.94 µm (Fig. 1b), whereas cell widths during passage exclusively in M9 glucose never exceeded 0.94 µm (Supplementary Fig. 1c). Thus, if cell shape is dictated by the medium they are in, cell width in M9 glucose would only decrease to below 0.94 µm. However, we observed that by 30 min after dilution into M9 glucose from a stationary-phase LB culture, cell width increased by ~8% and reached ~1.03 µm (Fig. 3c), a value that was not achievable for cells that were always passaged in M9 glucose (Supplementary Fig. 1c). Afterward, cell width dropped to ~0.9 µm, as expected for cells passaged in M9 glucose (Supplementary Fig. 1c), which we presume is mainly dictated by the M9 medium. Therefore, the initial widening of cells during outgrowth is not determined by external nutrients.

Outgrowth from stationary phase also involves changes such as altered expressions of stress-response genes, initiation of DNA replication, and different osmolalities of fresh versus stationary phase media. We tested these possibilities by repeating shape growth curve measurements in a ppGpp^0^ strain and a ∆*thyA* strain. The ppGpp^0^ strain is unable to synthesize ppGpp, a small nucleotide regulating stress-related genes^1^. The ∆*thyA* strain does not replicate DNA in the absence of external thymine^25^. In both strains, shape growth curves still exhibited similar outgrowth dynamics (Supplementary Note 3, Supplementary Fig. 1d–k). Systematically tuning the osmolality of media caused much smaller shape changes than those observed in the shape growth curves (Supplementary Note 3, Supplementary Fig. 1l). Taken together, we conclude that the initial increase in width is likely to be governed by proteome re-allocation rather than external factors or other biological processes.

A recent study showed that during steady-state growth, *E. coli* cells regulate surface area synthesis to maintain the ratio of *SA* and dry cell mass *M*^26^. Thus, we investigated whether the SA to dry mass ratio (SA/M) is similarly maintained during stationary-phase outgrowth. Using a microfluidic device, we monitored stationary-phase cells exposed to fresh medium and directly measured dry mass density using quantitative phase imaging (Methods). Dry-mass density was high in stationary phase, and gradually decreased during outgrowth by ~30%, consistent with the previous study^26^ (Supplementary Fig. 1m); these data indicate that density is not positively correlated with growth potential. Interestingly, SA/M first increased by 15%, and then gradually decreased (Supplementary Fig. 1n). Thus, SA/M is not maintained when cells grow out of stationary phase, indicating a different mode of surface area regulation during outgrowth from stationary phase. Noting that the density changes across growth phases alter the correlation between mass and volume, we focused our model on experimental measurements of cell volume.

### Cells exiting from stationary phase reach a critical SA/V at their first division

We next asked whether the initial increase of cell width was related to the first division after cells exiting stationary phase. We diluted stationary phase cells onto agarose pads containing fresh LB, tracked the growth of >200 individual cells until the first division occurred, and quantified their cellular dimensions over time. Cell width at division positively correlated with the initial width, with a slope of 0.52 ± 0.05 (mean ± s.e.m.). Therefore, cells did not divide at a critical cell width, nor did they widen by a fixed amount before division (Supplementary Fig. 2a), suggesting that cell width changes do not directly trigger the first division during stationary-phase outgrowth. Cell volume at division negatively correlated with the initial volume (Fig. 3d), indicating that the first division exiting stationary phase is not triggered by a critical volume or a critical added volume. Similar negative correlations were observed for surface area, cell length, and cross-sectional area (Supplementary Fig. 2b–d). Time to first division (Fig. 3e) and instantaneous growth rate at division (Supplementary Fig. 2e) both negatively correlated with initial cell volume, indicating that division does not occur after a fixed time interval or when cells reach a critical growth rate.

Previous work suggested that exponentially growing cells accumulate excess surface area material during the cell cycle, which then triggers division^23^. For a rod-shaped cell, division adds two hemispheric poles and increases SA by 4% without changing volume (Supplementary Fig. 2f). Since cells decrease their SA/V during outgrowth (Fig. 1d), we thus asked whether the first division after cells exit from stationary phase was also related to their SA/V. In our experiments, the SA/V at division was approximately constant and independent of the initial SA/V (Fig. 3f). Moreover, the normalized distributions of SA/V and cell width at division were much narrower than those of other cellular dimensions (Fig. 3g), indicating that a threshold value of cell width or SA/V could set the timing of the first division. Since cells do not reach a critical width at division (Supplementary Fig. 2a), the first division after stationary-phase exit is likely linked to a critical SA/V.

We further asked how division-related proteins were regulated during stationary-phase exit by tracking the dynamics of the key division protein FtsZ. We selected four *E. coli* strains, each containing a mutation in the actin homolog MreB that exhibited different mean widths and lengths during log-phase growth^11^. These mutants allowed us to analyze division dynamics in cells of different widths. These strains also contained a chromosomally integrated internal fusion of FtsZ to monomeric Venus (FtsZ^sw^-mVenus) at the native FtsZ locus^11,27^, allowing quantification of FtsZ abundance using fluorescence microscopy (Methods). All strains exhibited qualitatively similar cell shape dynamics as wild-type *E. coli*, despite their altered lengths and widths (Supplementary Fig. 2g). Total FtsZ fluorescence remained constant in the first 45 min post dilution before starting to increase (Fig. 3h), indicating that FtsZ synthesis started much later compared to other genes (Fig. 2e). We also measured FtsZ dynamics in a time-lapse experiment with GFP fused to the FtsZ promoter (P_*ftsZ*_-GFP)^24^ and confirmed that FtsZ expression started ~50 min post dilution (Supplementary Fig. 2h). Although the four MreB mutants had different levels of total FtsZ (Fig. 3h), FtsZ concentration was quantitatively similar across the different strains (Fig. 3h, inset), suggesting a conserved mechanism of FtsZ regulation independent of cell shape, consistent with previous measurements in exponential-phase cells^11^. In all strains, no FtsZ rings were observed until ~50 min post dilution. Virtually all cells contained one FtsZ ring by ~100 min post dilution (Fig. 3i), consistent with the observed timing of the first division after stationary-phase exit (Fig. 3e). Taken together, these data indicate that FtsZ levels are upregulated concurrent with the need for division.

### SA/V dynamics are broadly conserved across width and length perturbations

**Fig. 4:**
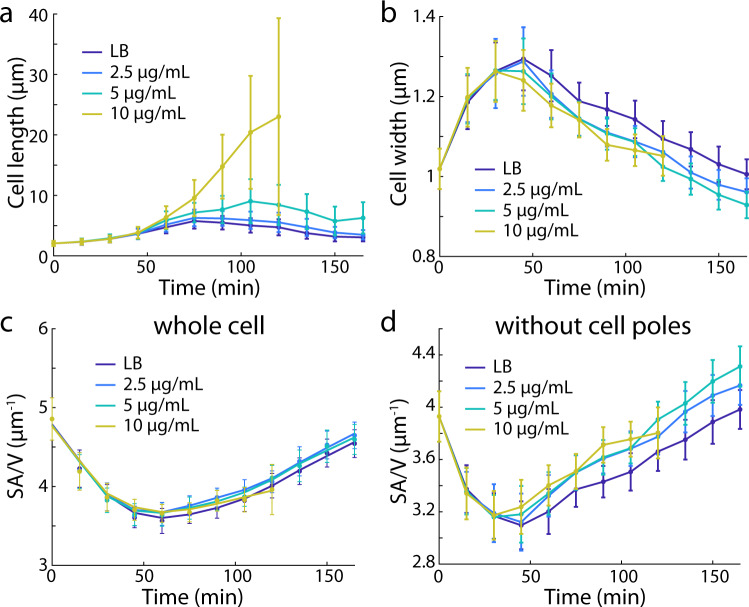
SA/V dynamics are conserved across perturbations to cell morphology. Shape growth curves of *E. coli* MG1655 cells treated with sub-lethal concentrations of cephalexin. Cephalexin causes a dose-dependent increase in cell length (**a**), accompanied by decreased cell width (**b**). The increased lengths and decreased widths precisely maintain SA/V for all conditions (**c**), suggesting that SA/V is robust to perturbations in cellular dimensions. The SA/V of the cell body (excluding the poles) was not maintained under cephalexin treatment (**d**), highlighting the importance of cell poles in regulating SA/V. Data points are mean ± S.D. with *n* > 200 cells. Source data are provided as a Source Data file.

Since SA/V is dependent on both cell width and length, we asked whether chemically or genetically tuning cell width or length would affect SA/V dynamics. We first treated wild-type *E. coli* cells with a range of concentrations of cephalexin, an antibiotic that inhibits the division-specific cell-wall synthesis enzyme PBP3^28^. Cell division increases SA/V by adding cell poles (Supplementary Fig. 2f). Importantly, lower cephalexin concentrations (2.5 µg/mL and 5 µg/mL) did not affect bulk growth rate for at least the first 10 h of growth. For the highest cephalexin concentration used (10 µg/mL), cells started to lyse after 2 h, but growth was not affected prior to lysis (Supplementary Fig. 3a). Thus, cephalexin treatment does not directly affect any parameters in our model, despite the obvious perturbations to cell length. Shape growth curves showed that cells became longer due to inhibition of cell division in a concentration-dependent manner (Fig. 4a). However, as cephalexin concentration was increased, cell width peaked at a lower value and began to decrease at an earlier time point (Fig. 4b). As a result, SA/V was maintained throughout the first 3 h (Fig. 4c) despite the marked changes in cell length. Furthermore, we quantified SA/V for the cell body alone by excluding the cell poles, and found that SA/V of the cell body varied with added cephalexin (Fig. 4d). Therefore, cell division has a measurable effect on SA/V by creating new cell poles (Fig. 4d). Taken together, cells collectively regulate width and length to maintain SA/V during growth, even when cell division is perturbed.

We further studied cell shape changes in a library of *E. coli* mutants with a wide range of mean cell lengths and widths^11^. While the strains had different morphologies, they all exhibited similar SA/V dynamics as observed in wild-type *E. coli* (Supplementary Note 4, Supplementary Fig. 3b–g), further indicating that SA/V dynamics (Fig. 1c) are conserved across genetic perturbations to cellular dimensions.

### SA/V dynamics are conserved across species and growth temperatures in rich media

**Fig. 5:**
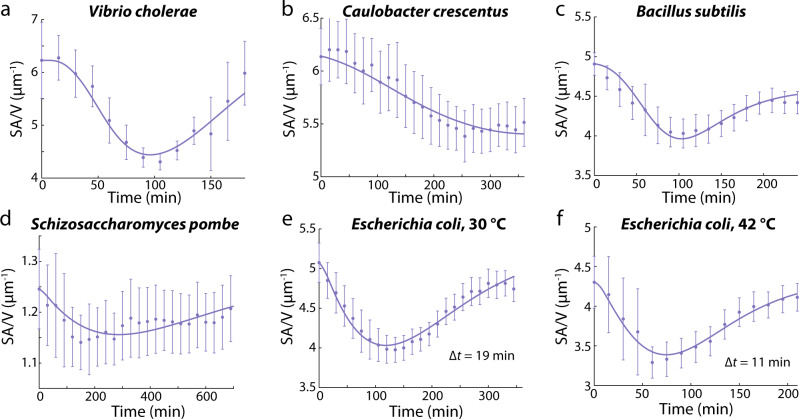
SA/V dynamics are conserved across species and growth temperatures. Shape growth curves for multiple species and temperatures. *V. cholerae* (**a**) and *C. crescentus* (**b**) are Gram-negative, *B. subtilis* (**c**) is Gram-positive, and *S. pombe* (fission yeast) is a eukaryotic fungi (**d**). In all cases, cells exhibited similar trends with SA/V decreasing when cells resumed growth, and then gradually recovering when cell growth slowed down again. Data points are mean ± S.D., with *n* > 40 for **d**, and *n* > 200 for all other panels. Solid lines are fits to the time-delay model. The time delay at 30 °C (**e**) was ~70% longer than that at 42 °C (**f**), consistent with the difference in steady-state growth rates. Source data are provided as a Source Data file.

The basis of our interpretation of SA/V dynamics during batch culture should be generally applicable to species other than *E. coli*, as it does not make any assumptions about *E. coli*-specific pathways. Hence, we predicted that a similar initial decrease in SA/V upon outgrowth from stationary phase should generally occur. We thus expanded our shape growth curve measurements to a variety of other species and conditions. We quantified shape growth curves for the Gram-negative bacteria *Vibrio cholerae* and *Caulobacter crescentus*, both of which form curved rods, the rod-shape Gram-positive bacterium *Bacillus subtilis*, and the eukaryote fission yeast *Schizosaccharomyces pombe*. In all species we tested, SA/V dynamics were similar to those in *E. coli*, with SA/V decreasing when cells resumed growth, and then gradually recovering when cell growth slowed down again (Fig. 5a–d, Supplementary Fig. 3h, Supplementary Note 5). Compared to the bacterial species, *S. pombe* exhibited a smaller change in cell width (~5%), presumably due to its thicker cell wall^29^. Such changes in cell width have been previously observed in *S. pombe* during nutrient depletion^30^. The SA/V dynamics in *S. pombe* (Fig. 5d) exhibited more fluctuations and deviated more from our model fitting than most bacterial species, suggesting that other mechanisms may also control *S. pombe* cell shape; nonetheless, both width and length changed along the growth curve in a manner similar to *E. coli*. Therefore, our time-delay model could apply to other microbial species, including eukaryotes.

Bacterial cell size during steady-state growth is thought to be relatively constant as growth rate changes across temperatures^5^. Nonetheless, we still found that *E. coli* cells at 30, 37, and 42 °C had different cellular dimensions and SA/V values. At all temperatures, cells still obeyed similar SA/V dynamics despite altered ∆*t* (Fig. 5e, f, Supplementary Note 5). Taken together, the initial decreases of SA/V appear to be general across microbial species, growth temperatures, and cell shapes and sizes, indicating that as cells accelerate in growth, volume synthesis always increases more quickly than surface synthesis.

### SA/V regulation is dependent on nutrient conditions

Since steady-state cell shape is nutrient-dependent^6^, we next asked whether medium composition affects SA/V dynamics. We quantified shape growth curves in M9 media supplemented with different carbon/nitrogen sources that support different growth rates. Overall, in low-nutrient conditions with slower growth rates, ∆*t* was close to zero and SA/V remained largely constant, despite substantial changes in cell width and length (Supplementary Note 6, Supplementary Fig. 4a–e). Richer nutrient conditions supported faster growth rates and led to larger changes in SA/V and non-zero ∆*t* values (Supplementary Note 6, Supplementary Fig. 4f–h). Since cell shape changes occurred even in defined media with a single carbon source (Supplementary Fig. 4c, d), the observed cellular dimension changes across shape growth curves are not likely due to diauxic shifts caused by nutrient consumption, but rather related to the dynamics of proteome composition as growth rate changes. A more complex panel of nutrients that support faster growth likely requires a larger shift in proteome composition (Fig. 2b), driving a larger range of SA/V changes (Supplementary Fig. 4i).

### Time-delay model quantitatively predicts SA/V decreases due to inhibiting cell-wall synthesis

**Fig. 6:**
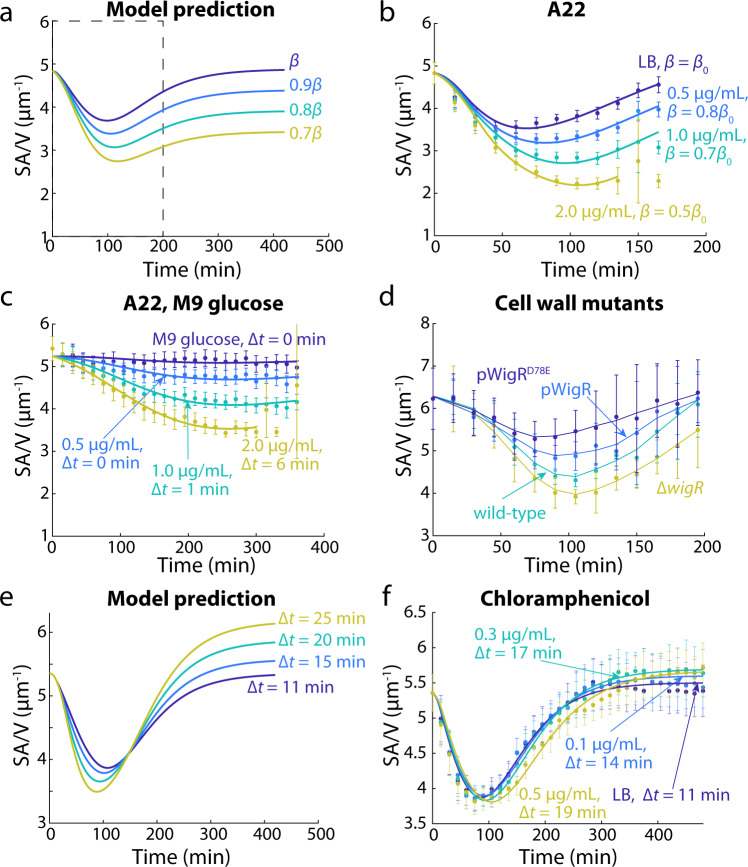
Time-delay model quantitatively predicts SA/V changes due to inhibiting cell-wall synthesis or protein translation. **a** The model predicts that decreasing *β* results in decreased SA/V over time. Dashed box highlights the time scale tested in experiment. **b** Shape growth curves during A22 treatment in LB exhibited similar SA/V dynamics as predicted in **a**. **c** Shape growth curves with A22 treatment in M9 glucose also exhibited decreases in SA/V, even though SA/V remained largely constant without A22. Higher A22 concentrations also led to non-zero ∆*t*, indicating that cells must adjust their proteome under inhibition of cell-wall synthesis. **d** Shape growth curves for *V. cholerae* cell-wall synthesis mutants. Overexpression of WigR (pWigR, pWigR^D78E^), which upregulates cell wall synthesis without affecting growth, increased SA/V. By contrast, ∆*wigR* cells have downregulated cell-wall synthesis and exhibited lower SA/V. **e** The time-delay model predicts that higher Δ*t* leads to lower SA/V in log phase, but higher SA/V when cells enter stationary phase again. **f** Shape growth curves of MG1655 cells treated with low levels of chloramphenicol. The dose-dependent SA/V dynamics were consistent with model predictions, and model fitting resulted in longer ∆*t* at higher doses. Data points in **b**, **c**, **d**, **f** are mean ± S.D., with *n* > 200 single cells in each condition. Solid lines in **b**, **c**, **f** are best fits to the time-delay model. Source data are provided as a Source Data file.

We next sought to test predictions of our time-delay model experimentally. In our model, reducing *β* (without changing *α*) led to the prediction that the shape growth curve would exhibit larger SA/V decreases during the first 2 h, with a final SA/V when cells returned to stationary phase lower than the initial value (Fig. 6a). The *E. coli* cytoplasm is surrounded by the cell envelope, constituted of three layers: the cell wall, and two membranes on either side of the cell wall. The cell wall mainly consists of peptidoglycan, while the membranes include lipids, proteins, and lipopolysaccharides. Since in most conditions the synthesis of peptidoglycan is more energetically costly compared to lipids^31^, we hypothesized that altering cell-wall synthesis would have a larger effect on *β*.

To validate our model prediction, we treated cells with multiple antibiotics that inhibit cell-wall synthesis and asked whether SA/V changes were consistent with our model predictions. We first treated MG1655 cells grown in LB with A22^32^, a small molecule inhibiting the actin homolog MreB and therefore affecting the spatial pattern of new cell wall incorporation. Sub-lethal concentrations of A22 did not affect growth rate during the first 3 h of growth, but cells treated with A22 exhibited dose-dependent increases in cell width compared to the non-treated control (Supplementary Fig. 5a), as previously shown^33^. Interestingly, the increase in cell width was accompanied by relatively lower increases in cell length (Supplementary Fig. 5b), which partially compensated for the decrease in SA/V. Together, the inhibition of cell-wall synthesis by A22 caused SA/V to drop in a dose-dependent fashion (Fig. 6b), showing the same trend as predicted by our model (Fig. 6a). Fitting the A22 shape growth curves to our model was indeed consistent with a dose-dependent decrease in *β* (Fig. 6b), while ∆*t* was not affected except at the highest A22 concentration, which resulted in a ~50% longer time delay (Supplementary Table 1), presumably because higher A22 concentrations require further proteome redistribution. We also tested two other cell-wall synthesis inhibitors, fosfomycin and mecillinam. Fosfomycin inhibits MurA, the enzyme catalyzing the first committed step of peptidoglycan biosynthesis^34,35^, and mecillinam specifically targets PBP2, a transpeptidase that crosslinks new cell-wall material^36^. In both cases, we observed similar changes to shape growth curves as with A22 treatment (Supplementary Fig. 5c, d).

Since cells grown in M9 glucose exhibited largely constant SA/V and virtually no time delay (Supplementary Fig. 4a), we asked whether inhibiting SA synthesis would affect their SA/V dynamics by treating cells grown in M9 glucose with A22. In this case, A22 also caused a similar drop in SA/V (Fig. 6c) and mean length (Supplementary Fig. 5e), and cell width now increased as opposed to the slight decrease in the A22-free control (Supplementary Fig. 5f). Thus, the relatively small range of SA/V changes observed in M9 glucose (Supplementary Fig. 4a) was indeed because the proteome composition for surface area and volume synthesis was largely balanced in M9 glucose. Nonetheless, perturbations such as A22 treatment can still break this proteome balance and alter SA/V more substantially. Fitting the A22 SA/V dynamics to our model also showed that at high A22 concentrations, in addition to decreased *β* (Supplementary Table 1), ∆*t* became non-zero (Fig. 6c), suggesting that cells have to re-allocate their proteome composition to accommodate inhibition of cell-wall synthesis.

Cell wall synthesis can also be altered genetically. In *V. cholerae*, activation of a histidine kinase/response regulator two-component system, WigK/WigR, increases expression of many cell-wall synthesis genes and elevates cell-wall synthesis^37^. We therefore quantified the shape growth curves of *V. cholerae* strains with a range of cell-wall synthesis capacities. Compared to wild-type, overexpression of WigR increased SA/V (Fig. 6d), mainly through decreased cell width. Overexpression of WigR^D78E^, a phosphomimetic version of WigR, further increases cell-wall synthesis^37^, and SA/V increased even more as expected (Fig. 6d), accompanied by further decreases in cell width. By contrast, deletion of WigR slows down cell wall synthesis, and we observed decreases in SA/V similar to those during treatment of *E. coli* with wall-acting antibiotics (Fig. 6d). Thus, genetically perturbing cell-wall synthesis affects *β* and therefore SA/V dynamics as predicted by our model.

We next tested whether inhibiting lipid synthesis would have similar effects on SA/V dynamics. While it has been previously shown that inhibiting fatty acid synthesis via treatment with cerulenin alters cell morphology^38^, those experiments were performed in a regime (cerulenin concentration >50 µg/mL) in which growth rates were strongly affected (Supplementary Fig. 5g). In the context of our model, at lower concentrations of cerulenin (<10 µg/mL) where growth rates remained unaffected (Supplementary Fig. 5g), we did not observe noticeable changes in cellular dimensions after 2 h of growth (Supplementary Fig. 5h). Similarly, in cell wall-deficient spheroplasts^39^, surface area was only limited by lipid synthesis, and those cells exhibited increased, rather than decreased, SA/V during outgrowth from stationary phase (Supplementary Note 7, Supplementary Fig. 5i). We further analyzed previously published proteome datasets^12,40^, and found that levels of lipid synthesis proteins, but not peptidoglycan synthesis proteins, increased monotonically with growth rate (Supplementary Note 7, Supplementary Fig. 5j–l). Taken together, lipid biosynthesis protein levels are likely directly linked to growth rate, and cell envelope growth is limited by cell wall rather than membrane synthesis.

### Inhibiting translation increases the time delay between volume and surface growth

The addition of the time delay ∆*t* was a critical modification to our model in order to fit our experimental shape growth curve data (Fig. 2f, Supplementary Fig. 1a). We therefore asked whether modifying Δ*t* would have observable effects on SA/V dynamics. By increasing Δ*t* from 11 to 25 min, our model predicted that the minimal SA/V reached in log phase would decrease, while the final SA/V when cells enter stationary phase would increase (Fig. 6e). Such non-monotonic changes to SA/V dynamics are somewhat counterintuitive, highlighting the biological relevance of the time delay. The initial SA/V drop after exiting stationary phase is primarily due to the quick increase in *α*. While *β* also increases, the time delay between *α* and *β* causes volume to grow faster than SA, leading to decreased SA/V. Therefore, a larger Δ*t* means that volume increases more before SA growth catches up, resulting in an even lower SA/V in log phase. As for the terminal SA/V, it is dependent on $\frac{{{\int} {{\mathrm{d}}SA/{\mathrm{d}}t} }}{{{\int} {{\mathrm{d}}V/{\mathrm{d}}t} }}$, an integral effect that depends on the entirety of the growth dynamics. $\frac{{{\mathrm{d}}V}}{{{\mathrm{d}}t}}$ only depends on *α* and remains unaffected, while $\frac{{{\mathrm{d}}SA}}{{{\mathrm{d}}t}} = \beta V\left( t \right) = f\left( {a\left( {t - \Delta t} \right)} \right)V(t)$. Increasing Δ*t* leads to increased ${\int} {{\mathrm{d}}SA/{\mathrm{d}}t}$ and eventually a higher terminal SA/V.

We tested these predictions by treating cells with low levels of chloramphenicol, a translational inhibitor, based on our inference that Δ*t* is mainly determined by rates of proteome re-allocation (Fig. 2b). Although inhibiting protein translation inevitably affected both *α* and *β*, at very low chloramphenicol concentrations (~0.01–0.05X minimal inhibitory concentration), cell growth was largely unaltered, and the SA/V dynamics matched our model predictions: increasing chloramphenicol concentrations increased ∆*t* by ~70%, while *β* only changed <5% across conditions (Fig. 6f, Supplementary Table 1). In these cases, the changes in SA/V were largely dictated by the relatively larger cell widths under chloramphenicol treatment (Supplementary Fig. 6a), as cell length followed very similar trends across chloramphenicol concentrations (Supplementary Fig. 6b). Higher chloramphenicol concentrations substantially reduced growth rate and led to even lower SA/V in log phase (Supplementary Fig. 6c). Model fitting found that the reduced SA/V was also mainly due to increased ∆*t*, as *β* changed less than 10% (Supplementary Table 1). Thus, our shape growth curves with chloramphenicol treatment provide further validation of our model predictions, and demonstrate that tuning Δ*t* has observable effects, rather than merely acting as a fitting parameter.

## Discussion

Although much work has been done to unveil the molecular players determining cell morphology at steady states, an approach unifying cell morphology and growth rates in dynamic environments is still lacking. In this study, our time-delay model of SA/V dynamics accurately predicted how cell size responds to growth rate changes during outgrowth from stationary phase (Supplementary Note 8), and was generally applicable across many species and growth conditions. The minimal assumption of a single, constant time delay between volume and surface area synthesis quantitatively recapitulated the complex SA/V dynamics (Figs. 1c and 5), and predicted SA/V changes under perturbations (Fig. 6). While previous studies have shown that cells adopt different steady-state SA/V values across growth conditions^6,23^, our work further reveals that the quantitative dynamics of SA/V under environmental perturbations are based on global resource allocation and the temporal dependence between surface area and volume growth (Fig. 2c–e). Our model thus has the potential to quantitatively describe other dynamic biological processes that scale with cell volume^41^.

While we obtained the time delay between surface area and volume synthesis (∆*t*) via model fitting, systematically tuning ∆*t* conferred non-monotonic changes in SA/V dynamics (Fig. 6e, f), highlighting its biological relevance. The changes between initial and saturated SA/V could potentially be used to infer how ∆*t* varies across conditions. We were able to measure the delay of surface area growth compared to volumetric growth by directly quantifying proteomic changes (Fig. 2d) and tracking protein expression in single cells (Fig. 2e). Such a time delay is consistent with previous studies of *E. coli* cells switched from one steady state to another^23^. Inhibiting protein translation increased ∆*t* (Fig. 6f, Supplementary Fig. 6c), suggesting that surface area synthesis was delayed due to a slower regulation of surface area synthesis-related enzymes. The connection between ∆*t* and changes in growth rate is complex, since higher growth rates may require larger shifts in proteome, but also speeds up proteome turnover. Thus, ∆*t* does not necessarily have a simple correlation with growth rates. Other factors, such as cell-wall precursor synthesis and insertion, could also alter ∆*t* in addition to proteome changes. It remains to be explored why and how surface area and volume synthesis rates are differentially modulated, and the ways in which such a delay can be beneficial for cells.

Our model predicts that upon growth resumption, cells should always increase in width, as surface area synthesis is limiting and increasing width rather than length poses a lower demand for surface area material (Fig. 3a). We observed such width increases after diluting stationary-phase cells into fresh media across all species and experimental conditions (Figs. 1b, d and 3c, Supplementary Figs. 3, 4 and 5). Similar changes in width have been reported in growth-inhibited cells due to the presence of an antibiotic, in which *E. coli* cells resumed growth after washing out the antibiotic and cell width increased prior to length increase^42^. The initial widening of cells is likely related to active growth, in which the transcriptional program favors synthesis of cytoplasmic proteins over envelope-related proteins (Fig. 2d, e), resulting in the subsequent imbalance between surface area and volume synthesis. In *E. coli*, such widening has been linked to altered cell wall insertion patterns governed by changes in MreB localization patterns^10,11^, providing a potential molecular mechanism in response to limited surface area synthesis. While DNA replication is a central process in cell proliferation and has implications in cell-size determination^6,43^, we have shown that cell shape remodeling can be independent of DNA replication (Supplementary Fig. 1f–k), at least in the initial 2 h of shape growth curves, indicating that the effect of DNA abundance on cell shape potentially manifests on a different time scale or is growth dependent. Regardless, our findings strongly suggest that the dynamic coordination between surface area and volume growth dictates cell shape.

*E. coli* cell division at steady state is well described by the adder model^44^. However, for cells growing out of stationary phase, we observed that cells did not add a constant volume, surface area, width, or length (Fig. 3d, Supplementary Fig. 2a–d). A recent study suggested that *E. coli* cells exhibit “sizer” behavior when exiting from stationary phase (first division at a critical size)^45^. While our data also showed near-“sizer” behavior (Fig. 3d, Supplementary Fig. 2b–d), we found that a critical SA/V was more likely to dictate the first division (Fig. 3f); the reported “sizer”-like behavior based on a critical volume is an approximation for the critical SA/V, with much lower coefficient of variation for SA/V (Fig. 3g). The SA/V at the first division was also more tightly regulated compared to cellular dimensions like length or volume (Fig. 3g). While cell width also exhibited a similarly narrow distribution as SA/V, division did not occur at a fixed cell width (Supplementary Fig. 2a). Moreover, before division occurred, cell width first increased and then decreased (Fig. 1a), and thus a width threshold for division is unlikely. The key division protein, FtsZ, was upregulated concurrently with the need for cell division (Fig. 3h, i, Supplementary Fig. 2h), consistent with previous work showing that a threshold of FtsZ is required for cell division^46,47^. The dynamics of FtsZ were further delayed compared to other cell-wall synthesis genes (Fig. 2e, Supplementary Fig. 2h), reinforcing the idea that gene expression during growth resumption is temporally regulated to allow cells to prioritize volumetric growth over surface area synthesis or division.

Our model of SA/V dynamics links cell width and length, even though the two dimensions are regulated by distinct molecular machineries^15,16,36^. Interestingly, perturbations known to increase cell width or length also led to decreases in the other dimension (Fig. 4a, b, Supplementary Fig. 5a, b, e, f). Larger cell width and length both decrease SA/V, and a corresponding decrease in cell length or width can compensate for an increase in the other to maintain SA/V. Therefore, despite the seemingly disjoint nature of cell width and length regulation in rod-shaped cells, the global resource limitation on surface area growth imposes constraints on the two dimensions. From an evolutionary perspective, bacterial cells constantly encounter feast or famine conditions, and therefore optimize the resource allocation strategies through regulations between surface area and volume growth. Our study mainly focused on *E. coli* cells, which are rod-shaped with growth occurring along the cell body. Nonetheless, our time-delay model does not explicitly model cell shape or growth patterns, and thus potentially applies to tip-growing and even non-rod-shaped cells. Rod-like shapes allow cells to efficiently tune SA/V via two disparate growth modes (Fig. 3a), which potentially confers evolutionary benefits compared to other shapes. In eukaryotic cells, a nuclear transporter receptor serves as a sensor of SA/V^48^, and it remains to be discovered whether similar global SA/V sensors exist in bacterial cells, which would provide opportunities to reveal new connections between cell physiology, size, and fundamental mechanisms of morphogenesis.

## Methods

### Strains and media

Strains used in this study are described in Supplementary Table 2. For routine culturing, all cells were grown in lysogeny broth (LB) at 37 °C unless otherwise specified. *C. crescentus* cells were grown in PYE (peptone-yeast extract) media at 30 °C^23^, and *S. pombe* cells were grown in YES255 media at 30 °C. Antibiotics (Sigma Aldrich, St. Louis, MO, USA) were used at the concentrations noted in the text. IPTG was used at a final concentration of 1 mM for *V. cholerae* cells. Thymine was added at a final concentration of 500 µg/mL for the *E. coli* Δ*thyA* strain. For minimal media, glucose or other carbon sources were added at a final concentration of 0.4%, six amino acids (L-methionine, L-histidine, L-arginine, L-proline, L-threonine, and L-tryptophan) were added at final concentrations of 500 µg/mL each, and casamino acids were added at a final concentration of 3% (w/v).

Strains were inoculated from freezer stocks into test tubes with 3 mL of media and supplemented with the appropriate antibiotics. The tubes were incubated overnight, except for cells grown in M9 acetate, which were incubated for 48 h. Overnight cultures were back-diluted 1:200 into the same fresh medium for shape growth curves and growth curve measurements.

Spheroplasts were grown in LFLB (LB with additional 3.6% sucrose and 10 mM MgSO_4_) at 30 °C, with 60 µg/mL cefsulodin added to inhibit cell wall growth^39^. For shape growth curves, overnight spheroplast cultures with cefsulodin were washed three times in fresh LFLB, and diluted 1:10 into LFLB with or without cefsulodin.

### Single-cell imaging

For experiments conducted on agarose pads, samples were taken from test tubes and placed on 1% agarose pads every 15 min, and then imaged within 5 min. For membrane staining, a small aliquot of cells was incubated with FM 4-64 (Invitrogen) at a final concentration of 5 µg/mL for 5 min and spotted on agarose pads without washing. Flow-cell experiments were performed in ONIX B04A microfluidic chips (CellASIC). Cells were loaded into the imaging chamber, and media reservoirs were filled with fresh or spent LB medium. Phase-contrast images and epifluorescence images were acquired with a Nikon Ti-E inverted microscope (Nikon Instruments) using a 100X (NA 1.40) oil immersion objective and a Neo 5.5 sCMOS camera (Andor Technology). The microscope was outfitted with an active-control environmental chamber for temperature regulation (HaisonTech, Taipei, Taiwan). Images were acquired using µManager v.1.4^49^.

### Population-level growth analyses

To measure growth dynamics, overnight cultures were inoculated into 200 µL of fresh media supplemented with the appropriate antibiotics in a clear 96-well plate. The plate was covered with an optical film, with small holes poked at the side of each well to allow aeration. Incubation and OD measurements were performed with an Epoch 2 plate reader (BioTek) at appropriate temperatures with continuous shaking and OD_600_ measured at 7.5-min intervals. The growth rate was calculated as the slope of ln(OD) with respect to time after smoothing using a moving average filter of window size five.

### Morphological analyses

The MATLAB (MathWorks, Natick, MA, USA) image processing code *Morphometrics*^20^ was used to segment cells and to identify cell outlines from phase-contrast or fluorescence microscopy images. A local coordinate system was generated for each cell outline using a method adapted from *MicrobeTracker*^50^. Cell widths were calculated by averaging the distances between contour points perpendicular to the cell midline, excluding contour points within the poles and sites of septation. Cell length was calculated as the length of the midline from pole to pole. Cell volume and surface area were estimated from the local meshing results. For calculations of cell volume and surface area, we assumed rotational symmetry about the inferred long axis of the cell.

### FtsZ fluorescence quantification

Cells with a sandwich fusion of mVenus to FtsZ^27^ were imaged in phase contrast and fluorescence using an ETGFP filter. FtsZ fluorescence was quantified by summing the intensity values of each pixel within the cell contour. FtsZ rings were identified as the peak of fluorescence intensity along the cell contour.

### *β* as a function of *α* at steady state

Based on previous steady-state growth rate and SA/V measurements, steady-state SA/V linearly correlates with growth rate^6^. Since steady-state SA/V equals *β*/*α*, and growth rate is *α*, *β*/*α* is a linear function of *α*. Therefore, based on SA/V measurements at two different growth rates, we can fit the function relating *β* to *α*. In our measurements in LB, *E. coli* cells approaching stationary phase correspond to *α* = 0 and *β*/*α* = 5.7 µm^−1^. By diluting cells repeatedly, cells reached steady-state morphologies^11^ for which *β*/*α* = 3.8 µm^−1^, and the corresponding *α* = 1.7 h^−1^. Thus, in LB, we have *β*/*α* = 5.7 – 1.12*α*, or *β* = *α*(5.7 – 1.12*α*).

### SA/V as a function of width and length changes

For calculations in Fig. 3a and Supplementary Fig. 2f, a cell with width *w* and length *l* was approximated by a cylindrical cell body with radius *r* = *w*/2 and length *l* *−* 2*r*, and hemispherical caps on each end with radius *r* *=* *w*/2. In this scenario, the corresponding surface area of the cell is *SA* *=* 2*πr*(*l* *−* 2*r*) *+* 2 × 2*πr*^2^ *=* *πwl*, and the volume is *V* *=* *πr*^2^(*l* *−* 2*r*) *+* 4*πr*^3^/3 = *πw*^2^*l/*4 *−* *πw*^3^*/*12. For the initial cell, *w* *=* 1 µm and *l* = 5 µm.

### Calculation of permissible range of *β*

In rod-shaped cells, given the above equations for *V* and *SA*, $\frac{{{\mathrm{d}}V}}{{{\mathrm{d}}t}} = \left( {\frac{\pi }{2}wl - \frac{\pi }{4}w^2} \right)\frac{{{\mathrm{d}}w}}{{{\mathrm{d}}t}} + \frac{\pi }{4}w^2\frac{{{\mathrm{d}}l}}{{{\mathrm{d}}t}} = \alpha \left( {\frac{\pi }{4}w^2l - \frac{\pi }{{12}}w^3} \right)$, and $\frac{{{\mathrm{d}}SA}}{{{\mathrm{d}}t}} = \pi l\frac{{{\mathrm{d}}w}}{{{\mathrm{d}}t}} + \pi w\frac{{{\mathrm{d}}l}}{{{\mathrm{d}}t}} = \beta \left( {\frac{\pi }{4}w^2l - \frac{\pi }{{12}}w^3} \right)$. During growth, $\frac{{{\mathrm{d}}w}}{{{\mathrm{d}}t}} \ge 0$ and $\frac{{{\mathrm{d}}l}}{{{\mathrm{d}}t}} \ge 0$. Solving for the linear optimization with the above constraints gives a permissible range of *β* that is dependent on *w*, *l*, and *α*, with minimum *β* corresponding to $\frac{{{\mathrm{d}}l}}{{{\mathrm{d}}t}} = 0$, and maximum *β* corresponding to $\frac{{{\mathrm{d}}w}}{{{\mathrm{d}}t}} = 0$.

### DAPI staining and fluorescence quantification

Cells were grown in the same conditions as in single-cell imaging experiments. At each time point, 1 mL of each sample was taken, pelleted at 6500*g* for 1 min, and fixed via resuspension in 500 µL 70% ethanol and incubation for 15 min at room temperature. Cells were then pelleted at 6500*g* for 1 min, resuspended in 500 µL PBS with 4′,6-diamidino-2-phenylindole (DAPI) added to a final concentration of 1 µg/mL, and incubated in the dark for 15 min. Cells were washed with PBS twice by pelleting at 6500*g* for 1 min followed by resuspension. Cells were spotted onto 1% agarose pads and imaged in phase contrast and fluorescence using a DAPI filter. DAPI fluorescence was quantified by summing the intensity values of each pixel within the cell contour, and then normalizing to the corresponding intensity in the control.

### qPCR

To estimate the relative replication rate of the chromosome, we quantified the copy numbers of 16 genetic loci via qPCR and fitted their log(relative abundance) to their corresponding distances to *terC*^19^. Cells were harvested at six different time points (from the overnight culture, and 30, 60, 90, 120, and 150 min after 1:200 dilution), and DNA was extracted using a DNeasy Blood & Tissue Kit (Qiagen). The relative abundances of chromosomal loci were quantified by qPCR, using the EvaGreen qPCR kit (Bio-rad). The qPCR probes used are listed in Supplementary Table 3.

### Proteome extraction

Overnight cultures were diluted 1:200 into LB and grown at 37 °C. During outgrowth from stationary phase, ~10^9^ cells were harvested and washed in cold PBS, then flash-frozen in liquid nitrogen. Two biological replicate samples were collected at 0, 15, 30, 45, 60, and 90 min during outgrowth. All samples were then thawed and resuspended in 500 µL lysis buffer (6 M urea, 50 mM Tris-base buffer, and 5% SDS) and subjected to 10 min of bead beating. After centrifugation, the supernatants were reduced with 10 µL of 500 mM DTT (Millipore Sigma) and alkylated with iodoacetamide (Millipore Sigma). The peptides were washed, digested, and eluted using S-trap tubes (Protifi) following the manufacturer′s protocols, desalted by C18 solid-phase extraction (Sep-Pak Waters), and dried by vacuum centrifugation. Peptide concentration was quantified for normalization using a Nanodrop ND-1000.

### Mass spectrometry proteomic analyses

Peptides were resuspended in 0.2% formic acid to a final concentration of 0.5 µg/µL. Subsequently, 1 µL was loaded onto an in-house laser-pulled 100-µm inner diameter nanospray column packed to ~22 cm with ReproSil-Pur C18-AQ 3.0 m resin (Dr. Maisch GmbH). Peptides were separated by reversed-phase chromatography on a Dionex Ultimate 3000 HPLC. Buffer A of the mobile phase contained 0.1% formic acid in HPLC-grade water, and buffer B contained 0.1% formic acid in acetonitrile. The HPLC used a two-step linear gradient with 4–25% buffer B for 135 min followed by 25–45% buffer B for 15 min at 0.400 µL/min. Peptides were analyzed on a LTQ Orbitrap Elite mass spectrometer (Thermo Fisher Scientific) in data-dependent mode, with full MS scans acquired in the Orbitrap mass analyzer with a resolution of 60,000 and *m/z* range of 340–1600. The top 20 most abundant ions with intensity threshold above 500 counts and charge states 2 and above were selected for fragmentation using collision-induced dissociation (CID) with an isolation window of 2 *m*/*z*, normalized collision energy of 35%, activation Q of 0.25, and activation time of 5 ms. The CID fragments were analyzed in the ion trap with rapid scan rate. Dynamic exclusion was enabled with repeat count of 1 and exclusion duration of 20 s. The AGC target was set to 1,000,000 and 50,000 for full FTMS scans and ITMSn scans, respectively. The maximum injection time was set to 250 ms and 100 ms for full FTMS scans and ITMSn scans, respectively.

### Peptide/protein database searching

Mass spectra were searched using Proteome Discoverer 2.2.0.388 using the built-in SEQUEST search algorithm. The Uniprot canonical *E. coli* FASTA database (4350 protein sequences downloaded on 2/9/2020) was used in this search, along with a database containing common preparatory contaminants. The precursor mass range was set to 350–3000 Da, the mass error tolerance was set to 10 ppm, and the fragment mass error tolerance to 0.6 Da. Enzyme specificity was set to trypsin, carbamidomethylation of cysteines (57.021) was set as variable modifications, oxidation of methionines (+15.995) and acetylation of protein N-terminus (+42.011) was considered as variable modifications. Percolator was used to filter peptides and proteins to a false discovery rate of 1%. Abundance quantification was based on precursor ion peak areas.

### Quantitative phase imaging and analysis

Stationary phase cells were loaded into the imaging chamber of an ONIX B04A microfluidic chip (CellASIC) initially with constant flow of spent LB. Cells were then exposed to fresh LB at the start of the time-lapse experiment. Images were acquired with a Ti-Eclipse inverted microscope (Nikon) with a 100X (NA: 1.4) DIC oil objective (Nikon) and a Zyla sCMOS 4.2 camera (Andor). μManager v. 1.41^49^ was used to automate acquisition of brightfield *z*-stacks with a step size of 100 nm from ±1 μm around the focal plane (total of 21 imaging planes) at 2-min intervals. At the end of the time-lapse, similar measurements were performed in LB supplemented with 50 mg/mL BSA to calibrate the phase shift equivalent to 50 mg/mL biomass.

For analysis, all imaging planes between +0.3 μm and −0.3 μm around the focal plane were used to calculate dry mass density^51^. Briefly, phase information was calculated based on intensity changes along the *z*-direction, and a Gaussian peak was fitted to the background of each image and then corrected to be at zero phase shift using an image-wide subtraction of the mean of the peak. The resulting images were segmented using *Morphometrics*^20^, and the intensity of pixels inside each cell was used to calculate the dry mass density.

### Reporting summary

Further information on research design is available in the Nature Research Reporting Summary linked to this article.

## Supplementary information

Supplementary Information

Description of Additional Supplementary Files

Supplementary Data 1

Reporting Summary

## Data Availability

The datasets generated and/or analyzed during the current study are available from the corresponding author on reasonable request. The mass spectrometry proteomics data have been deposited to the ProteomeXchange Consortium via the PRIDE^52^ partner repository with the dataset identifier PXD023729. Source data are provided with this paper.

## Source data

Source Data
