# Supplementary material for: Precise regulation of the relative rates of surface area and volume synthesis in bacterial cells growing in dynamic environments
Source: Nat Commun. 2021 Mar 30;12:1975. doi: 10.1038/s41467-021-22092-5 (PMC8009875; doi:10.1038/s41467-021-22092-5)
Supplement: Supplementary file 2 — Description of Additional Supplementary Files [file 41467_2021_22092_MOESM2_ESM.pdf]

Description of Additional Supplementary Files

File Name: Supplementary Data 1
Description: Proteome data at various time points after exit from stationary phase
