# Supplementary material for: Precise regulation of the relative rates of surface area and volume synthesis in bacterial cells growing in dynamic environments
Source: Nat Commun. 2021 Mar 30;12:1975. doi: 10.1038/s41467-021-22092-5 (PMC8009875; doi:10.1038/s41467-021-22092-5)
Supplement: Supplementary file 4 — Reporting Summary [file 41467_2021_22092_MOESM4_ESM.pdf]

nature research

Corresponding author(s): Kerwyn Casey Huang

Last updated by author(s): 01/24/2021

Reporting Summary

Nature Research wishes to improve the reproducibility of the work that we publish. This form provides structure for consistency and transparency in reporting. For further information on Nature Research policies, see our Editorial Policies and the Editorial Policy Checklist.

Statistics

For all statistical analyses, confirm that the following items are present in the figure legend, table legend, main text, or Methods section.

n/a	Confirmed	
☐	☒	The exact sample size (n) for each experimental group/condition, given as a discrete number and unit of measurement
☐	☒	A statement on whether measurements were taken from distinct samples or whether the same sample was measured repeatedly
☐	☒	The statistical test(s) used AND whether they are one- or two-sided<br>*Only common tests should be described solely by name; describe more complex techniques in the Methods section.*
☒	☐	A description of all covariates tested
☒	☐	A description of any assumptions or corrections, such as tests of normality and adjustment for multiple comparisons
☐	☒	A full description of the statistical parameters including central tendency (e.g. means) or other basic estimates (e.g. regression coefficient) AND variation (e.g. standard deviation) or associated estimates of uncertainty (e.g. confidence intervals)
☐	☒	For null hypothesis testing, the test statistic (e.g. F, t, r) with confidence intervals, effect sizes, degrees of freedom and P value noted<br>*Give P values as exact values whenever suitable.*
☒	☐	For Bayesian analysis, information on the choice of priors and Markov chain Monte Carlo settings
☒	☐	For hierarchical and complex designs, identification of the appropriate level for tests and full reporting of outcomes
☐	☒	Estimates of effect sizes (e.g. Cohen's d, Pearson's r), indicating how they were calculated

Our web collection on statistics for biologists contains articles on many of the points above.

Software and code

Policy information about availability of computer code

Data collection	Microscopy data were acquired using MicroManager v1.4. Growth curve measurements were acquired using the commercial BioTek Gen5 software v3.04.17.
Data analysis	Data analysis were performed using MATLAB R2018a. For single-cell analyses, the software packages Morphometrics v1.1 and Microbetracker v0.937 was used. Mass spectra were searched using Proteome Discoverer v2.2.0.388.

For manuscripts utilizing custom algorithms or software that are central to the research but not yet described in published literature, software must be made available to editors and reviewers. We strongly encourage code deposition in a community repository (e.g. GitHub). See the Nature Research guidelines for submitting code & software for further information.

Data

Policy information about availability of data

All manuscripts must include a data availability statement. This statement should provide the following information, where applicable:

- Accession codes, unique identifiers, or web links for publicly available datasets
- A list of figures that have associated raw data
- A description of any restrictions on data availability

The datasets generated during and/or analyzed during the current study are available from the corresponding author on reasonable request. The mass spectrometry proteomics data have been deposited to the ProteomeXchange Consortium via the PRIDE partner repository with the dataset identifier PXD023729. Source data are provided with this paper.

Field-specific reporting

Please select the one below that is the best fit for your research. If you are not sure, read the appropriate sections before making your selection.

- [x] Life sciences
- [ ] Behavioural & social sciences
- [ ] Ecological, evolutionary & environmental sciences

For a reference copy of the document with all sections, see nature.com/documents/nr-reporting-summary-flat.pdf

Life sciences study design

All studies must disclose on these points even when the disclosure is negative.

Sample size	No sample-size calculation was performed. For all single-cell imaging experiments, ~25 fields of view were acquired for each sample/ condition, yielding 200 - 1000 cells. Such a number of single cells is common for single-cell morphology studies.
Data exclusions	No data were exclude from the analyses.
Replication	Three biological replicates were included for growth curve measurements. Two biological replicates were included for mass spectrometry measurements. All attempts at replication were successful.
Randomization	For different treatment conditions, the same population of bacterial cells from a well-mixed overnight culture were aliquoted into parallel cultures that were treated by different conditions. Each aliquot contained ~10^7-10^8 single bacterial cells. The protocol is sufficient to create homogeneous treatment groups, and no further randomization was performed.
Blinding	All analyses pipelines were pre-programmed without tunable parameters that could skew the readout from each group from the researcher's input. Therefore, blinding was not relevant for this study.

Reporting for specific materials, systems and methods

We require information from authors about some types of materials, experimental systems and methods used in many studies. Here, indicate whether each material, system or method listed is relevant to your study. If you are not sure if a list item applies to your research, read the appropriate section before selecting a response.

Materials & experimental systems

n/a	Involved in the study	
x		Antibodies
x		Eukaryotic cell lines
x		Palaeontology and archaeology
x		Animals and other organisms
x		Human research participants
x		Clinical data
x		Dual use research of concern

Methods

n/a	Involved in the study	
x		ChIP-seq
x		Flow cytometry
x		MRI-based neuroimaging
